# Association of dietary magnesium intake with chronic constipation among US adults: Evidence from the National Health and Nutrition Examination Survey

**DOI:** 10.1002/fsn3.2611

**Published:** 2021-09-29

**Authors:** Lei Zhang, Zhang Du, Zhiang Li, Fei Yu, Lijun Li

**Affiliations:** ^1^ Department of Anorectal Surgery Dongyang People's Hospital Dongyang China

**Keywords:** bowel health, chronic constipation, magnesium, National Health And Nutrition Examination Survey

## Abstract

The association of dietary magnesium intake with chronic constipation has not been well‐studied in general population. Therefore, the aim of this study was to examine whether increased intake of dietary magnesium is associated with the presence of chronic constipation. Data from the 2007–2010 National Health and Nutrition Examination Survey (NHANES) were used. A total of 9,519 participants (4,814 men and 4,705 women) aged ≥20 years were included. The individual's bowel habits (chronic constipation) were evaluated using the questionnaire on bowel health and two different definitions of constipation (stool consistency and stool frequency) were used. Dietary magnesium intake was obtained from 24‐h dietary recall. Participants were categorized based on the quartiles of magnesium intake. Multivariable logistic regressions models were performed controlling for confounding factors. After multivariable adjustment, dietary magnesium intake was inversely associated with chronic constipation defined by stool frequency, and the ORs (95% CIs) across quartiles 2–4 compared with the lowest quartile were 0.71 (0.51–0.99), 0.78 (0.46–1.31), and 0.39 (0.16–0.95), respectively. In addition, there was a significant trend for the decreased prevalence of chronic constipation by quartiles of magnesium intake only among men (*p* for trend < .001). However, no statistically significant association between magnesium intake and prevalence of chronic constipation defined by stool consistency was observed. More evidence from longitudinal studies is needed to confirm these findings.

## Graphical abstract

This nationally representative study of Americans examined whether dietary magnesium intake was correlated with chronic constipation. The dietary intake of magnesium was inversely associated with the presence of chronic constipation defined by stool frequency in general population.

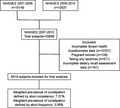

## 1 INTRODUCTION

Chronic constipation is one of the leading bowel conditions with estimated prevalence of 14% (95% confidence interval 12%–17%) in worldwide community‐dwelling populations (Suares & Ford, 2011). It is associated with impaired quality of life, high health care, and other indirect costs (Belsey et al., 2010). The causes of constipation are very complex and daily practice such as dietary factors and a sedentary lifestyle have a significant influence (Markland et al., 2013; Wilson, 2020). Hence, the modifications of lifestyle and diet are the first‐line recommendations for treatment of constipation, and diet is considered as a major modifiable lifestyle factor (Dupont & Hébert, 2020).

Magnesium is the fourth most abundant cation in the human body, and enzymatic databases list over 600 enzymes for which magnesium serves as a cofactor (de Baaij et al., 2015). And magnesium salts such as magnesium sulfate have been used as a treatment of constipation for their osmotic effects in the digestive tract (Vu et al., 2000). In recent, its positive effects have been proven in several double‐blind, randomized studies investigating the effects of sulfate‐rich mineral water on chronic constipation (Bothe et al., 2017; Naumann et al., 2016). However, the effect of magnesium intake from diet on chronic constipation in general population has rarely been studied (Murakami et al., 2007).

Epidemiological studies examining the effects of dietary factors on chronic constipation have utilized different definitions that include stool frequency, stool consistency, and the Rome Foundation Criteria (Markland et al., 2013; Shen et al., 2019; Yurtdaş et al., 2020). Previous studies showed that using different definitions (e.g., stool consistency vs. stool frequency) influences the estimated prevalence of constipation (Wilson, 2020). Consequently, it remains unclear whether the associations between dietary factors and constipation depend on how constipation is defined.

Given that epidemiological and clinical studies support an association of constipation with magnesium in the clinical setting (Bothe et al., 2017; Murakami et al., 2007), the primary aim of this study was to determine whether increased intake of dietary magnesium is associated with the presence of chronic constipation in general population. Secondary aim was to determine whether the associations between magnesium and constipation depend on how chronic constipation is defined.

## 2 MATERIALS AND METHODS

### 2.1 Study population

National Health and Nutrition Examination Survey (NHANES) is a cross‐sectional survey of a nationally representative sample of the noninstitutionalized population conducted by the National Center for Health Statistics (NCHS) of the Centers for Disease Control (Atlanta, GA, USA). Participants are noninstitutionalized individuals in the United States and are selected using a complex, stratified, multistage, probability cluster design. NHANES was approved by the National Center for Health Statistics’ Ethics Review Board and all participants provide written informed consent prior to completing the NHANES.

The current analysis has been limited to participants (age ≥20 years) from 2 cycles of NHANES (2007–2010) who completed the questionnaire on bowel health and dietary magnesium assessment. In total, 20,686 individuals participated in the NHANES during 2007–2010, and 10,359 participants completed the specific questionnaire on bowel health. Furthermore, participants who were pregnant (*n* = 108), take any laxatives (*n* = 571) or had no dietary data (*n* = 161) were excluded. Consequently, the final sample was comprised of 9,519 participants (4,814 men and 4,705 women).

### 2.2 Assessment of chronic constipation status

Participants were asked about bowel function during interviews including stool frequency and consistency within the last 30 days prior to the data collection time in the questionnaire on bowel health. One question dealt with stool frequency “How many times per week do you usually have a bowel movement?” In addition, participants were shown a colored picture card with descriptions of the seven Bristol Stool Form Scale types (BSFS; Type 1‐Type 7) and asked: “Please look at this card and tell me the number that corresponds with your usual or most common stool type.”

For this analysis, chronic constipation was defined in two different ways in order to compare the constipation definitions (Tab a et al., 2015; Wilson, 2020). The first definition for constipation was less than three bowel movements per week, while the second definition was based on participants reporting that their “usual or most common” stool type was either BSFS type 1 (separate hard lumps, like nuts) or BSFS type 2 (sausage‐like, but lumpy) as in other NHANES studies (Wilson, 2020).

### 2.3 Magnesium intake

The dietary intake of magnesium was assessed from a 24‐h dietary recall using the multipass recall approach. This approach is a respondent‐driven method of collecting an accurate and detailed list of all foods and beverages consumed by an individual during a 24‐h period (midnight to midnight). All participants were asked to participate in two 24‐h total nutrient recall interviews. The first 24‐h recall interview was done in person in the Mobile Examination Center (MEC), and the second 24‐h interview was conducted 3 to 10 days later through telephone. Therefore, if an individual completed both 24‐h recalls, we used the average magnesium intake from the two 24‐h recalls. Otherwise, we used the data from the first 24‐h recall. The distributions of magnesium intake were divided into quartiles in this study.

### 2.4 Covariates

A number of variables were evaluated as covariates hypothesized or previously shown to associate with constipation and magnesium intake (Markland et al., 2013; Tab a et al., 2015; Wilson, 2020). Sociodemographic characteristics included age (analyzed as continuous variable), gender (men and women), race/ethnicity (non‐Hispanic White, non‐Hispanic Black, and other race), and levels of education (≤high school, >high school). Behavioral risk factor assessments included smoking, alcohol consumption, and physical activity. Smoking status was classified as never smoker (never smoked or smoked <100 cigarettes in life), current smoker (smoked ≥100 cigarettes in life and currently smoking), or former smoker (smoked ≥100 cigarettes in life and currently no longer smoking). Participants who had at least 12 alcohol drinks per year were considered as drinkers. Physical activity was classified as vigorous physical activity (defined as “vigorous‐intensity activity that causes large increases in breathing or heart rate like carrying or lifting heavy loads, digging or construction work for at least 10 min continuously”) or no vigorous activity. Body mass index (BMI) was calculated as weight (kg) divided by height squared in m^2^ and categorized as under/normal weight (<25 kg/m^2^), overweight (25–29.9 kg/m^2^), and obese (≥30 kg/m^2^). Diabetes status was defined as being informed by doctor/health professional about the diagnosis of diabetes and/or a glycosylated hemoglobin measurement of ≥6.5% (Group, I. D. F. G, 2014). Patients with hypertension were defined as those who were taking medication for hypertension or informed by doctor/health professional about the diagnosis of hypertension, or whose systolic blood pressure exceeded 130 mmHg, or whose diastolic blood pressure exceeded 80 mmHg. To identify subjects with depression, the mental health questionnaire from the NHANES was used and the presence of depression was defined as a score of ≥10 on the Patient Health Questionnaire 9 (PHQ‐9), which is a 9‐item validated publicly available depression questionnaire (Kroenke et al., 2001). Dietary information on total fiber, fat, water and energy intake were assessed by trained interviewers based on the USDA Automated Multiple Pass Method.

### 2.5 Statistical analysis

Data were presented as mean ± standard deviation for continuous variables, and Analysis of Variance (ANOVA) was used to compare the mean levels across the quartiles of dietary magnesium intake. Number (percentage) was used for description of categorical variables and chi‐square tests were used to compare the distribution. Multivariate logistic regression analyses were conducted to examine the association between magnesium intake and risk of constipation adjusting for the above‐mentioned covariates, with quartile 1 as the referent category. Constipation was defined in two different ways based on stool consistency or frequency in this study. Odds ratio and 95% confidence interval (OR (95% CI)) were provided from multivariate logistic regression model. Tests for trend (*P*_for trend_) were performed by entering the magnesium intake (quartile‐categorical) as a continuous variable and rerunning the corresponding regression models. To detect effect modification of gender, separate analyses were conducted for men and women to investigate the association between magnesium intake and constipation.

Appropriate sampling weights provided by the NCHS were applied in the analyses to conduct a nationally representative estimate, taking into account the stratified, multistage probability sampling design. Stata 12.0 was used, and *p* < .05 was considered statistically significant.

## 3 RESULTS

### 3.1 Characteristics of the study sample

Tables 1 present participants’ characteristics. Of the 9,519 participants, 703 individuals reported chronic constipation defined by stool consistency (i.e., Bristol stool form score of 1 and 2) and 308 individuals reported chronic constipation defined by stool frequency (less than 3 bowel movements per week) (Table 1). The weighted prevalence of constipation defined by stool consistency and stool frequency were 7.01% and 3.08% in the overall sample, respectively. Participants with higher magnesium intake were predominantly men, younger, Non‐Hispanic White, and with a higher education level and percentage of vigorous physical activity practicing. In crude analyses, those with diets lower in magnesium were more likely to be constipated, no matter the definition of constipation (*p* < .001) (Table 1).

**TABLE 1:** Characteristics of study participants by quartiles of dietary magnesium intake in the 2007–2008 and 2009–2010 NHANES

Characteristics	Full sample, *N* = 9,519	Quartile 1, *N* = 2,385	Quartile 2, *N* = 2,381	Quartile 3, *N* = 2,382	Quartile 4, *N* = 2,371	*p Value*
Magnesium intake range (mg)	12.00–1358.00	≤ 199.50	199.50–264.00	264.00–349.50	≥349.50	
Age (years, mean ± *SD*)	49.60 ± 17.55	50.72 ± 18.75	50.45 ± 18.02	49.61 ± 17.11	47.58 ± 16.04	<.001
Gender, *n* (%)						<.001
Men	4,814 (50.57)	812 (34.05)	1,028 (43.18)	1,314 (55.16)	1,660 (70.01)	
Women	4,705 (49.43)	1573 (65.95)	1,353 (56.82)	1,068 (44.84)	711 (29.99)	
Race/ethnicity, *n* (%)						<.001
Non‐Hispanic White	4,630 (48.64)	1,023 (42.89)	1,112 (46.70)	1,218 (51.13)	1,277 (53.86)	
Non‐Hispanic Black	1789 (18.89)	643 (26.96)	477 (20.03)	375 (15.74)	303 (12.78)	
Other	3,091 (32.47)	719 (30.15)	792 (33.26)	789 (33.12)	791 (33.36)	
Levels of education, *n* (%)						<.001
≤high school	5,014 (52.73)	1549 (65.17)	1,311 (55.06)	1,132 (47.56)	1,022 (43.10)	
>high school	4,495 (47.27)	828 (34.83)	1,070 (44.94)	1,248 (52.44)	1,349 (56.90)	
Physical activity, *n* (%)						<.001
Vigorous physical activity	1866 (19.60)	266 (11.15)	390 (16.38)	502 (21.07)	708 (29.87)	
No vigorous activity	7,652 (80.40)	2,119 (88.85)	1991 (83.62)	1,880 (78.93)	1662 (70.13)	
Drinking status, *n* (%)						<.001
No	2,598 (27.31)	884 (37.10)	712 (29.92)	576 (24.20)	426 (17.97)	
Yes	6,916 (72.69)	1,499 (62.90)	1668 (70.08)	1804 (75.80)	1945 (82.03)	
Smoking status, *n* (%)						<.001
Never smoked	5,010 (52.64)	1,214 (50.90)	1,296 (54.45)	1,277 (53.61)	1,223 (51.60)	
Current smoker	2,151 (22.60)	662 (27.76)	505 (21.22)	470 (19.73)	514 (21.69)	
Former smoker	2,356 (24.76)	509 (21.34)	579 (24.33)	635 (26.66)	633 (26.71)	
Body mass index, *n* (%)						<.001
Under/normal weight	2,678 (28.36)	642 (27.24)	621 (26.29)	691 (29.19)	724 (30.73)	
Overweight	3,221 (34.11)	746 (31.65)	798 (33.78)	822 (34.73)	855 (36.29)	
Obese	3,543 (37.52)	969 (41.11)	943 (39.92)	854 (36.08)	777 (32.98)	
Diabetes, *n* (%)						<.001
No	8,068 (84.76)	1922 (80.59)	2007 (84.29)	2030 (85.22)	2,109 (88.95)	
Yes	1,451 (15.24)	463 (19.41)	374 (15.71)	352 (14.78)	262 (11.05)	
Hypertension, *n* (%)						<.001
No	4,456 (46.81)	1,020 (42.77)	1,061 (44.56)	1,113 (46.73)	1,262 (53.23)	
Yes	5,063 (53.19)	1,365 (57.23)	1,320 (55.44)	1,269 (53.27)	1,109 (46.77)	
Depression, *n* (%)						<.001
No	8,585 (90.55)	2038 (85.99)	2,146 (90.43)	2,194 (92.46)	2,207 (93.32)	
Yes	896 (9.45)	332 (14.01)	227 (9.57)	179 (7.54)	158 (6.68)	
Daily dietary intake (mean ± *SD*)						
Total fiber (g)	16.24 ± 8.76	8.62 ± 3.47	13.26 ± 4.09	17.61 ± 5.40	25.52 ± 9.72	<.001
Total fat (g)	75.71 ± 40.11	49.44 ± 22.05	65.84 ± 26.68	80.25 ± 31.22	107.50 ± 49.53	<.001
Total plain water (g)	899.59 ± 915.86	623.28 ± 685.01	875.69 ± 868.27	939.31 ± 895.67	1,161.62 ± 1,098.01	<.001
Total energy (kcal)	2040.85 ± 883.18	1,343.51 ± 466.50	1773.12 ± 505.53	2,168.22 ± 597.07	2,883.20 ± 1,005.62	<.001
Constipation defined by stool consistency						<.001
No	8,816 (92.61)	2,139 (89.69)	2,189 (91.94)	2,230 (93.62)	2,258 (95.23)	
Yes	703 (7.39)	246 (10.31)	192 (8.06)	152 (6.38)	113 (4.77)	
Constipation defined by stool frequency						<.001
No	9,211 (96.76)	2,253 (94.47)	2,304 (96.77)	2,314 (97.15)	2,340 (98.69)	
Yes	308 (3.24)	132 (5.53)	77 (3.23)	68 (2.85)	31 (1.31)	

### 3.2 Dietary magnesium intake and chronic constipation defined by stool consistency

When constipation was defined by stool consistency, the results of the multivariate logistic regression models are shown in Table 2. In the multivariate model, no statistically significant association between magnesium intake and prevalence of constipation was observed. The multivariate adjusted ORs (95% CIs) between magnesium intake and prevalence of constipation defined by stool consistency across quartiles 2–4 compared with the lowest quartile were 0.96 (0.69–1.32), 1.03 (0.74–1.43), and 1.13 (0.78–1.64), respectively.

**TABLE 2:** Multivariable‐adjusted logistic regression analysis of dietary magnesium intake associated with chronic constipation defined by stool consistency, NHANES 2007–2010

	Magnesium intake (mg)	Cases/participants	ORs (95% CIs)^a^
Overall (*n* = 9,519)			
Quartile 1	≤199.50	246/2385	1.00 (ref.)
Quartile 2	199.50–264.00	192/2381	0.96 (0.69–1.32)
Quartile 3	264.00–349.50	152/2382	1.03 (0.74–1.43)
Quartile 4	≥349.50	113/2371	1.13 (0.78–1.64)
*p* for trend			.500
Men (*n* = 4,814)			
Quartile 1	≤225.00	86/1210	1.00 (ref.)
Quartile 2	225.00–298.00	66/1205	0.91 (0.55–1.50)
Quartile 3	298.00–389.00	40/1197	0.67 (0.35–1.29)
Quartile 4	≥389.00	47/1202	1.36 (0.53–3.50)
*p* for trend			.837
Women (*n* = 4,705)			
Quartile 1	≤181.00	143/1180	1.00 (ref.)
Quartile 2	181.00–236.00	119/1178	0.78 (0.52–1.17)
Quartile 3	236.00–305.00	111/1173	0.85 (0.53–1.35)
Quartile 4	≥305.00	91/1174	0.83 (0.55–1.26)
*p* for trend			.508

^a^ Adjusted for age, gender (sex subgroup analysis excluded), race/ethnicity, levels of education, physical activity, drinking status, smoking status, body mass index, diabetes, hypertension, depression, total energy intake, total daily intakes of fat, fiber, and plain water.

### 3.3 Dietary magnesium intake and chronic constipation defined by stool frequency

When using stool frequency as the definition for constipation, the results of the multivariate logistic regression models are shown in Table 3. Dietary magnesium intake was inversely associated with constipation defined by stool frequency after multivariable adjustment, and the ORs (95% CIs) across quartiles 2–4 compared with the lowest quartile were 0.71 (0.51–0.99), 0.78 (0.46–1.31), and 0.39 (0.16–0.95), respectively (Table 3). The associations between magnesium intake and prevalence of constipation differed among men and women, and there was a significant trend for the decreased prevalence of constipation by quartiles of magnesium intake among men (*p* for trend < .001). However, the significant association was only observed when compared the second with the lowest quartiles of dietary magnesium intake among women (*p* for trend = .245).

**TABLE 3:** Multivariable‐adjusted logistic regression analysis of dietary magnesium intake associated with chronic constipation defined by stool frequency, NHANES 2007–2010

	Magnesium intake (mg)	Cases/participants	ORs (95% CIs)^a^
Overall (*n* = 9,519)			
Quartile 1	≤199.50	132/2385	1.00 (ref.)
Quartile 2	199.50–264.00	77/2381	0.71 (0.51–0.99)*
Quartile 3	264.00–349.50	68/2382	0.78 (0.46–1.31)
Quartile 4	≥349.50	31/2371	0.39 (0.16–0.95)*
*p* for trend			.090
Men (*n* = 4,814)			
Quartile 1	≤225.00	37/1210	1.00 (ref.)
Quartile 2	225.00–298.00	15/1205	0.31 (0.11–0.83)*
Quartile 3	298.00–389.00	19/1197	0.38 (0.19–0.75)**
Quartile 4	≥389.00	9/1202	0.10 (0.04–0.26)**
*p* for trend			<.001
Women (*n* = 4,705)			
Quartile 1	≤181.00	90/1180	1.00 (ref.)
Quartile 2	181.00–236.00	51/1178	0.59 (0.37–0.94)*
Quartile 3	236.00–305.00	53/1173	0.73 (0.41–1.30)
Quartile 4	≥305.00	34/1174	0.53 (0.20–1.42)
*p* for trend			.245

^a^ Adjusted for age, gender (sex subgroup analysis excluded), race/ethnicity, levels of education, physical activity, drinking status, smoking status, body mass index, diabetes, hypertension, depression, total energy intake, total daily intakes of fat, fiber, and plain water.

* *p* < .05

** *p* < .01.

## 4 DISCUSSION

In this large sample from the US general population, the associations between dietary magnesium intake and chronic constipation differed depending on the definition of chronic constipation. When using stool frequency as the definition for constipation, dietary magnesium intake was inversely associated with constipation.

Previous published studies showed varied prevalence estimates for constipation depending on the type of survey and the definition used to estimate the prevalence (Suares & Ford, 2011). In order to address some limitations of past epidemiological research on this topic, two different definitions of constipation (stool consistency and stool frequency) were used in our study. The prevalence of constipation defined by stool consistency were higher than a stool frequency‐based definition (7.01% vs. 3.08%, respectively) in this study. The results for prevalence of constipation were comparable to those from other studies on analyzing data from the NHANES database (Markland et al., 2013; Tab a et al., 2015).

There was evidence of an effect of magnesium salts such as magnesium‐rich natural mineral water on constipation symptoms in several randomized controlled studies (Bothe et al., 2017; Naumann et al., 2016). However, few studies have evaluated the association between dietary magnesium intake and constipation risk. In a cross‐sectional study on Japanese dietetic students aged 18–20 years, a low intake of magnesium from food was inversely associated with the prevalence of functional constipation (Murakami et al., 2007). In a recent cross‐sectional study including Japanese children aged 3 to 8 years, dietary intake of magnesium was not correlated with functional constipation (Fujitani et al., 2018). In our study, dietary intake of magnesium was inversely associated with constipation defined by stool frequency. The finding is biologically plausible. Studies evidenced that magnesium could individually exert a laxative action, which is mainly mediated by an osmotic effect due to their incomplete absorption in the gastrointestinal tract (Dupont & Hébert, 2020). In addition to the osmotic effect of magnesium, some mechanisms were also involved including a role of cholecystokinin and peptide YY endocrine secretions (Vu et al., 2000), increased expression of inducible nitric oxide synthase and antimicrobial action of magnesium (Uberti et al., 2020). In previous randomized controlled studies, the natural mineral water rich in magnesium sulfate improved both bowel movement frequency and stool consistency in subjects with functional constipation (Bothe et al., 2017; Naumann et al., 2016). However, no significant association between dietary intake of magnesium and stool consistency was observed in our study. Generally, data on stool size and consistency are more difficult to obtain, especially in epidemiology (Lackner et al., 2014; Weaver, 1988), which may impact the results on associations between dietary intake of magnesium and stool consistency. Even so, the differences of effect of dietary magnesium intake on stool consistency and stool frequency may involve other physiological mechanisms and more studies are needed. Further, sex differences have been noted when examining associations between magnesium intake and constipation. There was a significant trend for the decreased prevalence of constipation by quartiles of magnesium intake among men in our study, and the trend was not observed among women. Even sex differences have been noted in studies of constipation (Shen et al., 2019), and previous studies also observed the difference in reporting constipation symptoms and abnormal bowel habits among men and women at a tertiary referral center (McCrea et al., 2009). However, the physiologic mechanisms that underlie these gender differences warrant investigation.

Strengths of this study include use of a nationally representative sample of US adults with a large size and a wide array of demographic, dietary variables, and comorbidities available. Second, our results are also strengthened by the use of two different definitions of constipation (stool consistency and stool frequency). This investigation also has several notable limitations. First, causation cannot be determined because of the cross‐sectional nature of NHANES data. To minimize this limitation, participants who take any laxatives were excluded in our study. And information on dietary supplements of magnesium were not used in the calculation of dietary intake, which rendered it unlikely that dietary supplementation had a major impact on the findings. Second, both the bowel habit and dietary interview data were based on self‐reported measures. Generally, it is not possible to use more extensive or objective methods of assessing bowel habits or gastrointestinal function in large epidemiological studies such as NHANES.

In conclusion, the intake of magnesium was inversely associated with the presence of chronic constipation defined by stool frequency in general population, but not with constipation defined by stool consistency. More evidence from well‐designed longitudinal studies is needed to confirm these findings.

## CONFLICTS OF INTEREST

The authors declare that they have no conflicts of interest.

## ETHICAL APPROVAL

NHANES was approved by the National Center for Health Statistics Research Ethics Review Board.

## Data Availability

The data of this study can be available by contacting the corresponding author.
